# A benchmark database of ten years of prospective next-day earthquake forecasts in California from the Collaboratory for the Study of Earthquake Predictability

**DOI:** 10.1038/s41597-025-05766-3

**Published:** 2025-08-27

**Authors:** Francesco Serafini, José A. Bayona, Fabio Silva, William Savran, Samuel Stockman, Philip J. Maechling, Maximilian J. Werner

**Affiliations:** 1 School of Earth Sciences, University of Bristol, Bristol, UK; 2 Statewide California Earthquake Center, University of Southern California, Los Angeles, USA; 3 Nevada Seismological Laboratory, University of Nevada Reno, Reno, USA

**Keywords:** Natural hazards, Databases

## Abstract

Short-term seismicity forecasting models are increasingly developed and deployed for Operational Earthquake Forecasting (OEF) by government agencies and research institutions worldwide. To ensure their reliability, these forecasts must be rigorously tested against future observations in a fully prospective manner, allowing researchers to quantify model performance and build confidence in their predictive capabilities. The Collaboratory for the Study of Earthquake Predictability (CSEP) operated twenty-five fully automated M ≥ 3.95 seismicity models developed by nine research groups from Italy, California, New Zealand, the United Kingdom, and Japan. Between August 2007 and August 2018, these models produced over 50,000 daily forecasts for California, each specifying expected earthquake rates on a predefined space-magnitude grid over 24-hour periods. In this article, we describe the forecast database, summarize the underlying models, and demonstrate how to access and evaluate the forecasts using the open-source pyCSEP Python toolkit. The forecast data are publicly available through Zenodo, and the pyCSEP software is openly available on GitHub. This unprecedented dataset of fully prospective earthquake forecasts provides a critical benchmark for developing and testing next-generation OEF models, fostering advancements in earthquake predictability research

## Background & Summary

Short-term earthquake forecasting systems, and in general Operational Earthquake Forecasting^1^ (OEF) models, are currently operationalised in several countries, providing institutions, decision makers and citizens with statements on future seismicity in time spans of days to weeks that can ultimately support risk mitigation actions. These systems are at the foundation of all major OEF activities, and we should ensure that they incorporate the best science available. To this end, earthquake forecasting models should be evaluated against future seismicity in a fully prospective fashion using fair, reproducible, and statistically rigorous methodologies. The Collaboratory Study for Earthquake Predictability^2,3^ (CSEP) is a global community of researchers with the aim of organising fully prospective, transparent and reproducible earthquake forecasting experiments around the globe to advance earthquake predictability and enhance our understanding of the earthquake generation process. CSEP provides strict definitions of the test regions, evaluation periods, magnitude (*M*) ranges, as well as the datasets and testing procedures used to evaluate seismicity models. This is made available to the wider community through the Python toolkit pyCSEP^4–6^, an open-source software package designed to access and process earthquake catalogs, manage and evaluate probabilistic earthquake forecasts against observed data, and provide visualization tools and other utilities. In this way, CSEP offers a consistent theoretical framework and software implementation, with which one can evaluate forecasting models against observed seismicity.

One of CSEP’s major multi-institutional accomplishments was the development and operation of twenty-five seismicity models that provided daily forecasts of seismicity for California between August 1, 2008, and August 30, 2018, resulting in a database of over 50,000 forecasts. These forecasts were autonomously produced in the context of the Regional Earthquake Likelihood Models^7–12^ (RELM) initiative at the W. M. Keck CSEP testing Center^13^ in California. Having a monolithic testing center provided advantages such as ensuring a fully prospective design by controlling the data used by the models, fairness by providing all models with the same data and computational resources, and transparency as the models were not operated by the modellers themselves. This approach has been successful for over a decade; however, advancements in open science and open-source software have led us to enhance this format.

CSEP released the pyCSEP toolkit in September 2022, which provides researchers with a software library to evaluate and interact with CSEP forecasts. More recently, CSEP released a software application called floatCSEP^14,15^ that provides the complete experiment definition, data, rules and model artifacts as source code, which can be readily curated in open-data repositories for third parties to download and run to fully reproduce the experiment results. We are making this database of forecasts available to download so it can be used as a benchmark problem for developing future models and novel testing metrics.

The aim of this article is to describe the database of next-day forecasts, the computational tools to evaluate and compare forecasts, and how they are accessed through Zenodo, Github and the CSEP website. Along with the forecasts produced by each model in HDF5 format, we also provide Python code to analyse the forecasts, as well as a tutorial (in Python Jupyter notebook format) on how to use the code and learn pyCSEP. Specifically, we provide code to (i) load the forecasts in a pyCSEP-friendly format for specific sets of dates, (ii) create cumulative forecasts covering periods of multiple days, (iii) explore the temporal, spatial, and magnitude distributions provided by the forecast, (iv) use CSEP tests to assess consistency of forecasts and observations, and to compare alternative models, v) provide guidance on how to interpret the results.

Here, we show an example of analysis by focusing on the ETAS and STEP models’ forecasts throughout 2010, the year of the *M* 7.2 El Mayor-Cucapah earthquake. This analysis can be easily reproduced and expanded by considering new forecasting models, or new testing metrics. Researchers can use this database as a common point of comparison for new forecasting models, evaluate them with the code we provide, keep track of the improvements of their models over time, or, alternatively, assess the ability of new tests in distinguishing between models in the database given that the differences between them are known. Moreover, having this large set of forecasts is important to identify periods and areas where all the models perform poorly which, in turn, may provide insights on what needs to be improved by future models. The fact that the forecasts were created in controlled conditions with zero degrees of freedom ensures, along with the various applications for this database, makes it an excellent candidate to be a benchmark earthquake forecasting problem with the aim of improving future forecasting models

## Methods

The seismicity forecasts that make up the database described in this article, as part of the RELM initiative, were produced with a common experimental design, and compiled by scientists working at the W. M. Keck CSEP testing Center^13^ at the Statewide California Earthquake Center (SCEC). More specifically, all the models produced forecasts in the same format, for the same region, within the same depth and magnitude boundaries, and using the same input earthquake catalogue to increase comparability. To guarantee transparency and reproducibility, all forecasts were produced at the CSEP testing center^13^ following the same workflow. Research groups participating in the experiment provided computer code to produce the forecasts, the code was installed at the testing center, checked for stability and consistency, and then operated on a daily basis independently from the modellers. Each forecast is produced only with observations up to the corresponding day and, therefore, they are fully prospective. In this Section, we provide a description of the experimental design and a brief description of each model.

### Experimental design

**Fig. 1:**
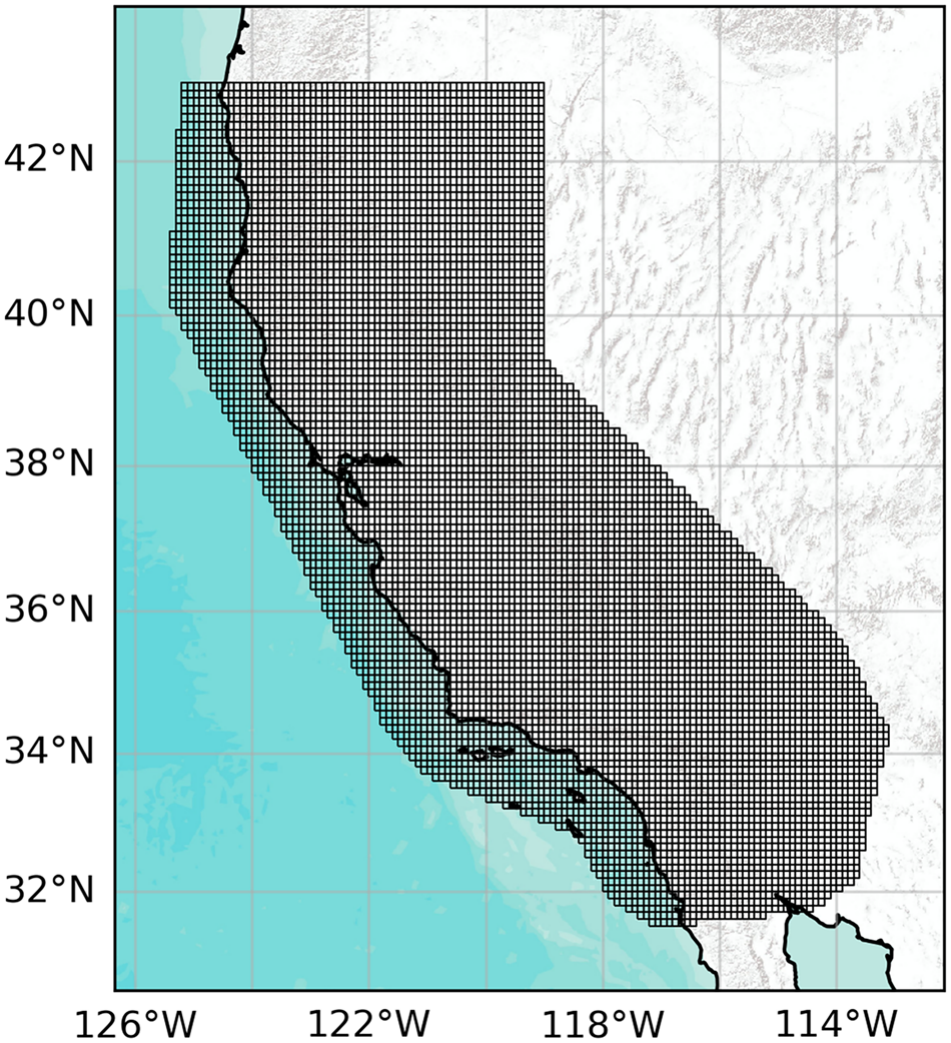
Space grid used for California used to produce the forecasts and to evaluate them. The region is provided within the pyCSEP toolkit.

The experimental design and the corresponding forecasts’ format are the same as those used for the Regional Earthquake Likelihood Models [7–12, RELM] initiative. Each daily forecast is in grid-based format^16^, and is represented by the expected rate (number of earthquakes) per bin in a prespecified space-magnitude grid in a given depth range (0, 30 km). The geographical region is covered by a 0.1° × 0.1° grid reported in Fig. 1. Similarly, the magnitude intervals are composed by 0.1 magnitude units bins from 3.95 to 8.95, with a larger open-end bin for events with magnitude above 8.95. The value 8.95 was adopted as a conservative estimate of the maximum possible magnitude in California which is estimated to be between 8 and 8.5^17,18^. Therefore, each cell is composed by a two dimensional spatial bin and a one dimensional magnitude bin. Each forecast starts at 00: 00: 00 UTC, ends at 23: 59: 59 UTC, and only earthquakes that have occurred before the starting time of the forecast can be used as input by the models. The models cover the period from August 1, 2008 to August 30, 2018. In this period, 571 *M* ≥ 3.95 earthquakes with depths less than 30 km were reported by the ANSS Comprehensive Earthquake Catalog^19^ (ComCat). Notable large earthquakes and aftershock sequences include, for example, the 2010 *M* 7.2 El Mayor-Cucapah^20,21^ seismic sequence, the 2012 *M* ≥ 4.0 Brawley earthquake swarm, and the 2014 *M* 6.0 South Napa^22,23^ earthquake.

The tests provided by pyCSEP for this forecast format are mostly likelihood-based tests. They have been introduced to evaluate earthquakes forecast by Schorlermmer^16^ and Zechar^24^, which defined tests to assess (in)consistencies between the number, spatial, and magnitude distributions of observed and expected earthquakes. Werner *et al*.^25^ introduced the conditional likelihood test to assess the overall consistency between forecasts and observations. All of them assume that, in each space-magnitude bin, the number of events follows a Poisson distribution with parameter equal to the rate specified by the forecast. For comparing forecasts, pyCSEP provides access to the information gain proposed by Rhoades^26^, and the Kagan information score^27^. Models use different earthquake catalogues for calibration as described in the corresponding references (see Table 1), and their parameters are not updated during the experiment. However, the same earthquake catalogue is provided to models as input data, and is used for evaluating the prospective forecasts; this is the ComCat catalogue.

**Table 1:** Summary information of the 25 CSEP models that produced next-day seismicity forecasts.

Name	Start Date	End Date	Missing days	File dimension	Calibration Period	(*M*_0_, *M*_*c*_)
ETAS^31^	01/08/2007	30/08/2018	0	5.8 GB	1898–1984	(3.95, ∞)
ETASV1.1^31^	01/07/2012	30/06/2018	4	3.19 GB	1898–1984	(3.95, ∞)
ETAS-DROneDayMd3^33^	01/10/2012	30/06/2018	5	2.66 GB	1932–2004	(3, 8)
ETAS-DROneDayMd2^33^	01/10/2012	30/06/2018	17	2.72 GB	1932–2006	(2, 8)
ETAS-DROneDayMd2.95^33^	01/07/2016	30/06/2018	18	930 MB	1932–2004	(2.95, 8)
ETASSYN-DROneDayMd2.95^33^	01/07/2016	30/06/2018	11	924 MB	1932–2004	(2.95, 8)
ETAS-HWMd2^25^	01/10/2012	19/09/2016	0	1.36 GB	1981–2012	(2, 8)
ETAS-HWMd3^25^	01/10/2012	30/06/2018	5	1.97 GB	1985–2012	(3, 8)
GSF-ISO^38^	01/10/2016	30/06/2018	1	726 MB	1981–2005	(2.5, ∞)
GSF-ANISO^38^	01/10/2016	30/06/2018	1	331 MB	1981–2005	(2.5, ∞)
STEP^39^	01/08/2007	31/01/2013	0	1.78 GB	1992–2006	(3.95, ∞)
STEPJAVA^39^	01/09/2010	30/06/2018	0	2.39 GB	1992–2006	(3.95, ∞)
KJSSOneDayCalifornia^42^	01/01/2009	30/06/2018	0	4.17 GB	1932–2008	(3.95, 8.05)
KJSSFiveYearsCalifornia^42^	01/10/2012	30/06/2018	15	2.44 GB	1932–2008	(3.95, 8.05)
K3Md2^44^	01/10/2012	19/09/2016	0	1.55 GB	1981–2012	(2, 8)
K3Md3^44^	01/10/2012	30/06/2018	5	2.24 GB	1985–2012	(3, 8)
ETAS-DROneDayPPEMd2^34^	01/10/2012	30/06/2018	18	2.70 GB	1932–2006	(2, 8)
ETAS-DROneDayPPEMd3^34^	01/10/2012	30/06/2018	6	2.70 GB	1932–2004	(2, 8)
JANUSOneDay^33^	01/10/2012	30/06/2018	18	2.50 GB	1932–2004	(2.95, 8.05)
JANUSOneDayEEPAS1F^33^	01/10/2012	30/06/2018	17	2.45 GB	1932–2004	(2.95, 8.05)
JANUSOneDayPPE^33^	01/10/2012	30/06/2018	17	2.23 GB	1932–2004	(2.95, 8.05)
JANUSOneDayTV^33^	01/10/2012	30/06/2018	17	982 MB	1932–2004	(2.95, 8.05)
ETAS-HW-K3-AVERAGE-Md2^44^	01/10/2012	19/09/2016	1	1.81 GB	1981–2012	(2, 8)
ETAS-HW-K3-AVERAGE-Md3^44^	01/10/2012	30/06/2018	23	2.61 GB	1985–2012	(3, 8)
SE2OneDay^52^	01/10/2012	30/06/2018	15	2.44 GB	1984–2004	(4.95, 8.05)

The table reports the model name with reference to the publication where to find more detailed information, the start and end of the forecasting period, the number of missing days, the file dimension, the period used to train (calibrate) the model, the minimum (*M*_0_) and corner (*M*_*c*_) magnitudes. The models are divided in three groups: ETAS models (top), alternative to ETAS models (middle), ensemble models (bottom).

### Model descriptions

**Fig. 2:**
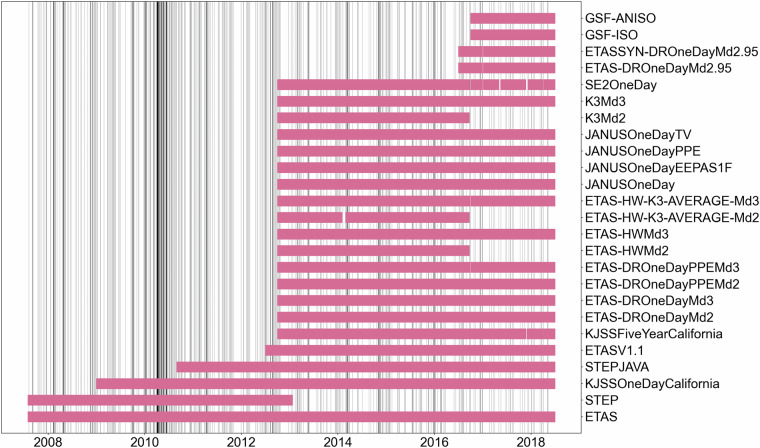
Models’ coverage: each row represents the days for which the corresponding model have produced forecasts, gaps represents missing days. Vertical lines represent occurrence of earthquakes in the testing region with *M*_*w*_* ≥ *3.95.

The next-day CSEP forecast database comprises 25 different models covering different periods between August 1, 2008 and August 30, 2018. Figure 2 shows the period covered by each model. Majority of models started producing forecasts in October 2012, most of them continuing until August 2018, while only few cover the period 2008–2012. Table 1 reports exact starting and end date of each model along with additional information, including the references, the number of missing days (days for which the model does not provide a forecast, mainly due to technical problems, see below), the dimension of the file, the period used to calibrate the model, and the magnitude range.

The models can be divided in three groups: different versions of the *Epidemic-Type Aftershock Sequence*^28^ (ETAS) model, alternatives to the ETAS model, and ensemble models. Table 2 summarises the modelling choices made for the first two groups of models, detailing how each model represents background seismicity, time dependence, spatial and magnitude distributions of events. We can appreciate that most models differ in terms of background and spatial components, while there is more homogeneity regarding the temporal and magnitude components. Regarding the latter, most of the models consider a form of the Gutenberg-Richter (GR) law^29,30^ frequency-magnitude distribution. Table 3 illustrates ensemble models and their components.

**Table 2:** Additional information of the ETAS and alternative to ETAS models.

Name	Background	Temporal triggering	Spatial triggering	Magnitude distribution
ETAS^31^	Spatial smoothing kernel	Omori law	Isotropic power law with magnitude bandwidth	Standard GR law
ETASV1.1^31^	Spatial smoothing kernel	Omori law	Isotropic power law with magnitude bandwidth	Standard GR law
ETAS-DROneDayMd3^33^	Spatio-temporal PPE^34^ model	Omori law	Isotropic Gaussian with magnitude bandwidth	Tapered GR law (*M*_*c*_ = 8.05)
ETAS-DROneDayMd2^33^	Spatio-temporal PPE^34^ model	Omori law	Isotropic Gaussian with magnitude bandwidth	Tapered GR law (*M*_*c*_ = 8.05)
ETAS-DROneDayMd2.95^33^	spatio-temporal PPE^34^ model	Omori law	Isotropic Gaussian with magnitude bandwidth	Tapered GR law (*M*_*c*_ = 8.05)
ETASSYN-DROneDayMd2.95^33^	spatio-temporal PPE^34^ model	Omori law	Isotropic Gaussian with magnitude bandwidth	Tapered GR law (*M*_*c*_ = 8.05)
ETAS-HWMd2^25^	spatial adaptive kernel estimator^36^	Omori law	Anisotropic^37^ if *M* ≥ 5.5, isotropic otherwise	Tapered GR law (*M*_*c*_ = 8.0)
ETAS-HWMd3^25^	spatial adaptive kernel estimator^36^	Omori law	Anisotropic^37^ if *M* ≥ 5.5, isotropic otherwise	Tapered GR law (*M*_*c*_ = 8.0)
GSF-ISO^38^	Spatial smoothing kernel	Omori law	Nonparametric isotropic funcion	Standard GR law
GSF-ANISO^38^	Spatial smoothing kernel	Omori law	Nonparametric function of the fault strike angle	Standard GR law
STEP^39^	Model by Frankel *et al*.^40^	Model by Reasenberg and Jones^41^	Model by Reasenberg and Jones^41^	Standard GR law
STEPJAVA^39^	Model by Frankel *et al*.^40^	Model by Reasenberg and Jones^41^	Model by Reasenberg and Jones^41^	Standard GR law
KJSSOneDayCalifornia^42^	Spatial smoothing kernel	Omori law	Isotropic Rayleigh distribution	Standard GR law
KJSSFiveYearsCalifornia^42^	Spatial smoothing kernel	—	—	Standard GR law
K3Md2^44^	Adaptive smoothing kernel	Adaptive smoothing kernel	Adaptive smoothing kernel	Adaptive smoothing kernel
K3Md3^44^	Adaptive smoothing kernel	Adaptive smoothing kernel	Adaptive smoothing kernel	Adaptive smoothing kernel
ETAS-DROneDayPPEMd2^34^	Spatio-temporal PPE^34^ model	—	—	Tapered GR law (*M*_*c*_ = 8.05)
ETAS-DROneDayPPEMd3^34^	Spatio-temporal PPE^34^ model	—	—	Tapered GR law (*M*_*c*_ = 8.05)

The columns of the table report (left to right) the name of the model, the type of model used for the background observations, the type of temporal triggering function, the type of spatial triggering function, and the magnitude distribution. The models are divided in two groups: ETAS models (top) and alternative to ETAS models (bottom).

**Table 3:** Additional information of the ensemble models.

Name	Models in the ensemble
JANUSOneDay^33^	Convex linear combinarion of PPE model^34^, EEPAS model^34^, and ETAS-DROneDayMd model^33^
JANUSOneDayEEPAS1F^33^	Convex linear combinarion of PPE model^34^, EEPAS model^34^, and ETAS-DROneDayMd model^33^
JANUSOneDayPPE^33^	Convex linear combinarion of PPE model^34^, EEPAS model^34^, and ETAS-DROneDayMd model^33^
JANUSOneDayTV^33^	Convex linear combinarion of PPE model^34^, EEPAS model^34^, and ETAS-DROneDayMd model^33^
ETAS-HW-K3-AVERAGE-Md2^44^	Convex linear combination of K3Md2 model^44^ and ETAS-HWMd2 model^25^
ETAS-HW-K3-AVERAGE-Md3^44^	Convex linear combination of of K3Md3 model^44^ and ETAS-HWMd3^25^
SE2OneDay^52^	Linear combination of EEPAS model^34^ and STEP model^39^

The table reports the name of the model (left) and the name.

All the models are calibrated on earthquakes from the ComCat catalogue, the only differences are in the time period and magnitude range. Below, we provide a brief description of the models specifying only the data used for calibration (implying the ComCat catalogue is used). The models are sorted by group: ETAS models first, alternatives to ETAS second, and ensemble models third.

#### ETAS models

- **ETAS, ETASV1.1**: These versions of the ETAS model were introduced by Zhuang^31^. They assume a spatially varying background rate, the Omori’s law for the temporal triggering function, and an isotropic power-law kernel with bandwidth depending on the magnitude of the event as spatial triggering function. The spatially varying background rate is obtained by smoothing the epicenters classified as background events. The magnitude distribution is a standard GR law with cutoff magnitude 3.95. The only difference between ETAS and ETASV1.1 is the smoothing kernel, which has the same function, but the latter has smaller bandwidth (less smooth). The parameters of the model are calibrated following the algorithm proposed by Zhuang^32^ with more than 4, 700 earthquakes with 3.95 ≤ *M* ≤ 6.0 from January 1, 1898 to January 1, 1985.
- **ETAS-DROneDayMd2, ETAS-DROneDayMd3, ETAS-DROneDayMd2.95, ETASSYN-DROneDayMd2.95**: These versions of the ETAS model were introduced by Rhoades^33^. The models employ a spatio-temporally varying background rate given by the Proximity to Past Earthquakes (PPE) model^34^, the Omori law as temporal triggering function, and an isotropic Gaussian kernel with magnitude dependent bandwidth as spatial triggering function. The magnitude distribution follows a Tapered GR law^35^ with cutoff magnitude 2.95 and corner magnitude 8.05. The main difference between the models is the catalogue used to calibrate the parameters. ETAS-DROneDayMd2 was fitted to an input catalog of *M* ≥ 3.95 earthquakes from 1932 to 1987 and *M* ≥ 1.95 from 1988 to 2006. The model ETAS- DROneDayMd3 was fitted on *M* ≥ 3 earthquakes, while ETAS-DROneDayMd2.95 and ETASSYN-DROneDayMd2.95 on *M* ≥ 2.95 earthquakes from 1932 to 2004. Another difference is that ETAS-DROneDayMd2, ETAS-DROneDayMd3, ETAS-DROneDayMd2.95 only accounts for the first generation of aftershocks, while ETASSYN-DROneDayMd2.95 accounts also for subsequent generations.
- **ETAS-HWMd2, ETAS-HWMd3**: These versions of the ETAS model were introduced by Werner *et al*.^25^. The models employ a time-independent background rate varying over space given by the adaptive smoothing method by Helmstetter^36^, the Omori law as temporal triggering function, an isotropic spatial triggering function for events with *M* < 5.5, and an anisotropic one for *M* ≥ 5.5. The anisotropic spatial triggering function is obtained by smoothing the locations of early aftershocks^37^. The magnitude distribution is a tapered GR law with cutoff magnitude 2.0 and corner magnitude 8.0 and *b* = 1. The value *b* = 1.72 is considered for earthquakes in the Geysers region in northern California. The only difference between the two models is the data used to calibrate the parameters. ETAS-HWMd2 was calibrated on earthquakes between January 1 1981 and March 1 2012 with magnitude *M* ≥ 2.0, while ETAS-HW-Md3 was calibrated over the same time and space period to *M* ≥ 3.0 earthquakes. Both models use a correction factor accounting for spatio-temporal variations in magnitude incompleteness.
- **GSF-ISO, GSF-ANISO**: These models are described by Gordon *et al*.^38^. They are nonparametric instances of ETAS with an inhomogeneous spatial triggering function based on the idea that aftershocks are more likely to occur along the local strike angle of the fault. The triggering function is estimated nonparametrically, while the local strike angle at any point in time and space is estimated using microseismicity. The difference between the models is that GF-ISO assumes an isotropic spatial triggering function, while GF-ANISO assumes this is anisotropic. The components of the models are estimated nonparametrically using eqarthquakes with *M* ≥ 2.5 between January 1, 1981 and August 23, 2005.

#### Alternative models

- **STEP, STEPJAVA**: These models are two instances of the Short-term Earthquake Predictability^39^ (STEP) model. Similarly to ETAS, the STEP model also is composed of a background and aftershock (or cluster) component but there are two crucial differences. Firstly, the STEP model assumes cluster-specific parameters for the magnitude and space-time distributions of aftershocks, and model parameters for each cluster are adjusted as new earthquakes accrue to it. Secondly, the background and aftershock components are not combined by summing them but by taking the maximum. The model by Frankel *et al*.^40^, which then underpinned the US national seismic hazard model, is used as background model, while the model by Reasenberg and Jones^41^ is used for the aftershock component. The difference between STEP and STEPJAVA is that the STEP model was implemented in MatLab while STEPJAVA in Java. This is because the MatLab version was deprecated, and the Java version was installed as replacement. Both models are calibrated on *M* ≥ 4.0 seismicity recorded from January 1, 1993 to November 1, 2006.
- **KJSSOneDayCalifornia, KJSSFiveYearCalifornia**: These models were introduced by Kagan and Jackson^42^. The KJSSFiveYearCalifornia model is obtained from a long-term model determined by smoothing the location of past seismicity, while the KJSSOneDayCalifornia model uses the branching model by Kagan and Jackson^43^ for the aftershocks. The latter assumes an Omori law decay in time and isotropic spatial triggering function, and a spatially varying background rate obtained by smoothing the locations of observed earthquakes using an isotropic kernel given by a Rayleigh distribution. The magnitudes follow a standard GR law with *b* = 0.975. The parameters are calibrated using 4, 497 *M* ≥ 4.0 earthquakes between January 1, 1932 and October 2, 2008. The difference between the two models is that KJSSOneDayCalifornia includes a fraction of the KJSSFiveYearCalifornia model plus time-dependent contributions from all previous earthquakes.
- **K3Md2, K3Md3**: These models were introduced by Helmstetter and Werner^44^. Differently from the other models, they rely on the use of adaptive smoothing kernels to estimate the space, time, magnitude distribution of earthquakes. Therefore, they do not make use of the ETAS formulation, the Omori’s law, or the GR law, and constitute a non-parametric alternative to the other models. The difference between the two models resides in the data used to calibrate them. The K3Md2 model uses earthquakes with *M* ≥ 2.0 recorded from January 1, 1981 to March 3, 2012, while the K3Md3 model uses earthquakes with *M* ≥ 3.0 recorded between January 1, 1985, and March 3, 2012.
- **ETAS-DROneDayPPEMd2, ETAS-DROneDayPPEMd3**: These are the PPE^34^ background model components of ETAS-DROneDayMd2 and ETAS-DROneDayMd3, respectively. Comparisons of each ETAS-DROneDay model with its PPE background model component will be useful in measuring how much additional information is provided by the aftershock triggering component.

#### Ensemble models

- **JANUSOneDay, JANUSOneDayEEPAS1F, JANUSOneDayPPE, JANUSOneDayTV**: These models were introduced by Rhoades^33^. The JANUSOneDay model is a linear combination of three models: the PPE^34^ model representing the background or long-term seismicity; an Every Earthquake a Precursor According to Scale^34,45^ (EEPAS) model representing medium-term seismicity, and the aftershock component of the ETAS-DROneDayMd models representing short-term seismicity. The parameters of each model component were calibrated on *M* ≥ 2.95 earthquakes from 1932 to 2004, while the weights of the linear combination are optimised using earthquakes that occurred from 1985 to 2004.
- **ETAS-HW-K3-AVERAGE-Md2, ETAS-HW-K3-AVERAGE-Md3**: These models by Helmstetter and Werner^44^ are obtained as convex linear combinations of K3Md2 and ETAS-HWMd2, and, respectively, of K3Md3 and ETAS- HWMd3. The idea behind this approach is that combining a parametric model, ETAS, with a non-parametric one, K3Md*, should combine their strengths. The models are combined by estimating a weighted average of the forecasted rates in each space-magnitude bin provided by two models. The weights were determined from the same data used to estimate the parameters of each model in the ensemble.
- **SE2OneDay**: This model is obtained by a linear combination of the EEPAS^34^ model with weight equal to 0.42 and the STEP^39^ model with weight 0.52. The magnitude distribution is a standard GR law with *b* = 0.91. The parameters and the weights of the SE2OneDay model were calibrated on 152 *M* ≥ 4.95 earthquakes between January 1, 1984 and September 30, 2004.

## Data Records

The next-day grid-based forecast database for California is openly and freely available for download from Zenodo^53^ (https://zenodo.org/records/15076187) and through the CSEP website (https://cseptesting.org/grid-based-forecasts On Zenodo, we provide all the forecasts generated by the models, the code to evaluate the forecasts against observations and to compare alternative models using pyCSEP, and the tutorial showing how to use the code in a single zip folder. Forecasts derived from single models can be downloaded from the CSEP website.

In both cases, the forecasts produced by a model are stored in the Hierarchical Data Format version 5^46^ (HDF5) file format. This hierarchical format allows to store multiple files divided in groups (similarly to a system of folders). In our case, each HDF5 file represents a model and contains multiple tables presenting the daily forecasts. The forecasts provided by a model are grouped in a system of nested folders with two levels. The first level represents the year, the second level represents the months, and, inside each month folder, we have one file for each day in which the model has issued a forecast. HDF5 files can be accessed using the tutorial we provide, which relies on the h5py python library^47^, or with online tools (e.g. https://myhdf5.hdfgroup.org).

For each day, the forecast is provided as a table in CSV file format. This table represents the rate expected by the model in each bin of a pre-defined space-magnitude grid (see Fig. 1 for the spatial region). Each row of the table represents a bin in the space-magnitude grid (there are exactly 391, 782 cells), while the columns represent the longitude and latitude extremes of the spatial bins (always with length 0.1°), the depth range (always from 0 to 30 km), the extremes of the magnitude bins (all with length 0.1 units), the expected rate of earthquakes, and a flag representing a legacy value from CSEP testing centers that indicates whether a bin should be evaluated or not, for a total of 10 columns.

## Technical Validation

The forecasts provided in this database underwent four validation phases. The first three validation phases were performed at the Testing Center^13^, while the last was performed independently by researchers at the University of Bristol on the finalised database. The first validation step was done before the installation of each model, and assessed the reliability of the code provided by model developers. The other two phases were executed during operations. The last phase aimed to check for missing or duplicate days and assessed the overall coherence of the results provided by each model.

Zechar *et al*.^13^ describe the validation steps performed at the Testing Center, as well as all activities performed, including a typical day of operations. The first validation step ensured that the modellers’ code compiles correctly, and that externally generated forecasts were identical to those generated at the Testing Center (to a small tolerance level). This phase also verified that the forecast generation was stable enough to be automated. To maximise system stability, the Testing Center was composed of three identical machines. New models were installed, debugged, and validated on the development machine. Models were then moved to the certification machine where they were tested for stability and consistency over periods of weeks to months. This was done by a suite of automated acceptance tests in which the modellers provided a set of inputs and the corresponding expected outputs. The outputs were compared with the expected ones provided by the modellers, and tolerance was allowed to account for machine precision errors. After this step, models were installed on the third machine to automatically generate the next-day forecasts. Relevant model inputs and metadata was stored to increase the transparency and reproducibility of the results. The second and third validation phases involved continuous validation of the workflow during operations to find issues when computing the forecasts. Sporadically, issues arose in processing due to problems in accessing the earthquake catalog or in executing model code. In these cases, manual reprocessing of the files was performed which did not impacted the prospective nature of the forecasts.

**Fig. 3:**
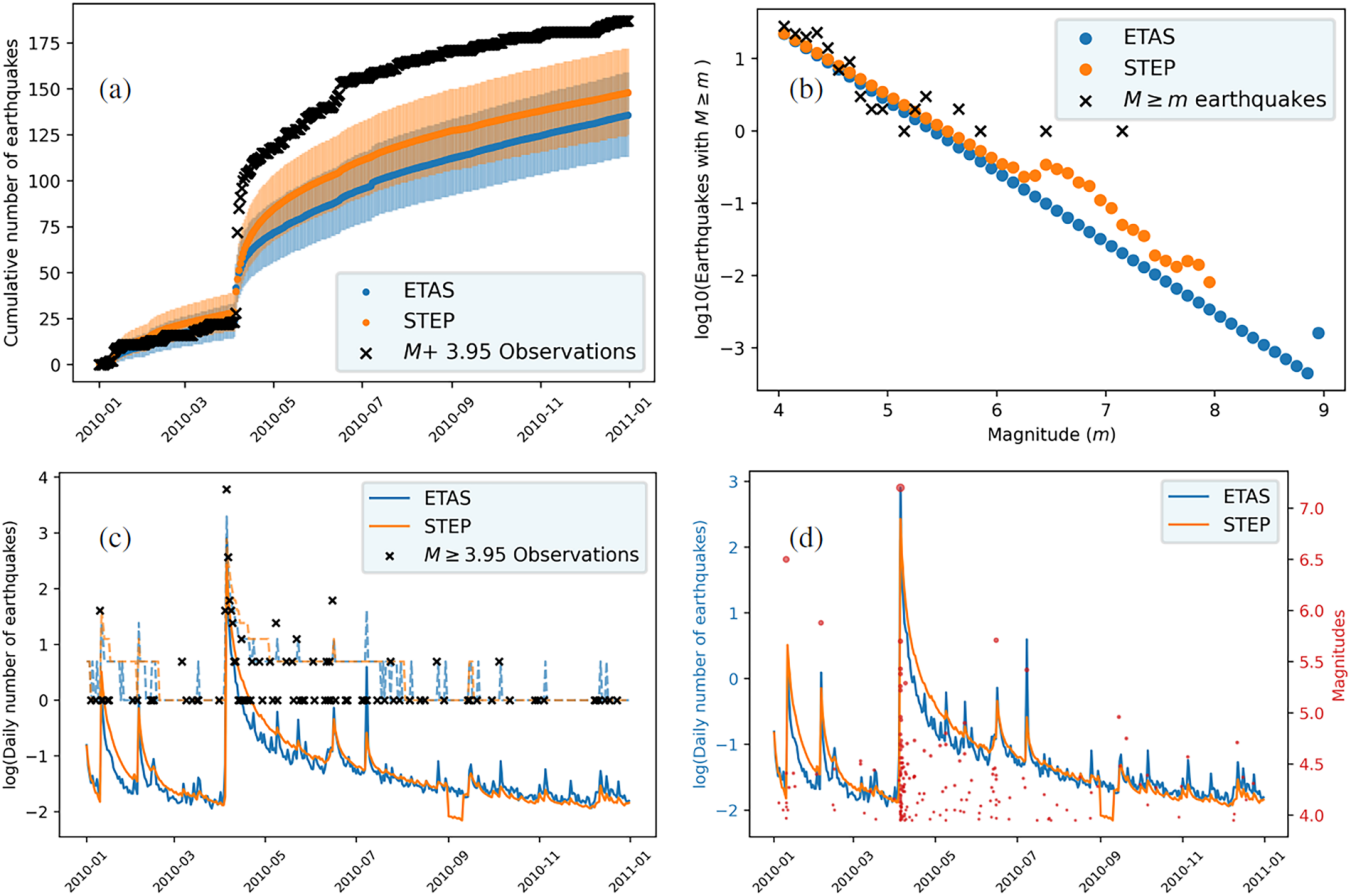
Descriptive analysis. Panel (**a**) Cumulative number of *M* ≥ 3.95 earthquakes expected by the ETAS^31^ (blue) and STEP^39^ (orange) along with the observed numbers (black cross). The shaded region represents Poisson uncertainty. Panel (**b**) Incremental magnitude distribution represented by the logarithm in base 10 of the number of events with magnitude greater or equal than *m* for varying values of *m*. Blue points represent the ETAS model, orange points represent the STEP model, and black crosses represent the observed catalog. Panel (**c**) Logarithm of the absolute number of *M* ≥ 3.95 earthquakes per day. The blue line represents the ETAS model, the orange line represents the STEP model, and black crosses represents the observed catalogue. Dashed lines represent the upper end of the 95% confidence interval of the logarithm of the number of earthquakes calculated considering a Poisson distribution for the number of earthquakes. Panel (**d**) Logarithm of the absolute number of *M* ≥ 3.95 earthquakes expected by the ETAS (blue) and STEP (orange) models, red dots represent observed magnitudes.

The last validation step was performed independently from the Testing Center and assessed the presence of duplicate and missing days, and the overall coherence of the forecasts provided by different models. This was needed as the previous validation phases may have led to files being stored in the wrong place (creating duplicates and gaps). Three models (ETASV1.1, STEP, and STEPJAVA) had one duplicate date, while one model (ETAS) had two. Given that the duplicate forecasts provided different information (different number of events, and spatial distribution) we investigated the consistency with forecasts corresponding to days before and after the duplicate day by looking at the temporal evolution of the total number of earthquakes expected by the model. If by visual inspection there was no clear favorite, we kept the most recently generated forecast. The majority of models have some missing days for which there is no forecast. Table 1 provides the number of missing days for each model. Lastly, we analysed the temporal evolution of the number of earthquakes expected by each model as shown in Fig. 3 for 2010 (panel a, c, and d). The aim is to check if there are unexpected jumps, a symptom indicating something went wrong while producing the forecast. The analysis confirmed that there are no unexpected jumps, the expected numbers of earthquakes provided by the models are smooth and the vertical jumps correspond with earthquake clusters, as expected.

## Usage Notes

In addition to the HDF5 forecast files, we also provide computer code written in Python, along with a tutorial, to load, visualise, evaluate the forecasts, and to convert forecasts in CSV file format to HDF5. This facilitates the use of this database as benchmark for future models. Indeed, researchers can produce new forecasts with their models, convert them into HDF5 files, and use the code we provide to analyse them. The code heavily relies on the pyCSEP^4–6^ toolkit to visualise the forecasts’ spatial distribution, compute CSEP consistency and comparison tests, and download the ComCat earthquake catalogue used to evaluate the forecasts.

In this Section, we describe how the database and provided software can be effectively used to establish a benchmark problem. Specifically, we illustrate these tools by focusing on the ETAS and STEP models’ forecasts throughout 2010, the year of the *M* 7.2 El Mayor-Cucapah earthquake, the strongest event in the period covered by the forecasts. The tutorial shows how to create all the figures and results reported in this article, and can be easily adapted to analyse new models as long as they provide an HDF5 file. The tutorial and code are openly accessible as part of the zip file on Zenodo^53^, namely the tutorial_hdf5.ipynb and functions_for_hdf5.py files, they can also be accessed separately from the CSEP website (https://cseptesting.org/grid-based-forecasts/).

### Benchmark

This database contains next-day seismicity forecasts, provided in a standardised format, generated from 25 models. This collection of forecasts can be used as a benchmark for evaluating new models and new testing metrics. Having a benchmark is crucial to bolster innovation because it enables one to efficiently compare different modelling approaches while ensuring consistency and reproducibility of the results. Consistency comes from the fact that the forecasts in the database were generated with zero degrees of freedom, i.e. defined on the same region, the same time intervals, and updated using the same observations. The only freedom is in the earthquake catalogue used to fit the model and determine the parameters values used afterwards to produce forecasts. Reproducibility comes from the fact that results obtained using the code we provide will be fully reproducible (except for machine errors). This is particularly true if this code is used in combination with the floatCSEP package. In this way, strengths and weaknesses of new models and metrics can be identified and put into context of existing modelling approaches. Indeed, comparing new models with models in this benchmark allows one to quantitatively measure their performance and be ranked accordingly. Similarly, the database can be used to evaluate different ensemble modeling techniques. To reuse the code to analyse new forecasts it is essential that the forecasts are stored in a HDF5 file. We provide functions to convert a set of forecasts organised in a system of nested folder in a HDF5 file. The only requirement is that the forecasts need to be organised in folders following a year/month/day hierarchy. This structure is described in details in the tutorial and we also provide an example forecast to be used as template.

Secondly, having a set of models with known characteristics (and differences) is important for developing new testing metrics. This is because it offers a standardised problem to assess properties of new metrics like their ability to distinguish between models, how different two models must be for the test to say so, which aspects of a forecast are penalised the most and which ones the least, and similar questions. This is essential to identify aspects of the forecast that are not accounted by CSEP tests, or to understand how this is taken into consideration, and design new ones, or provide alternatives to the current ones. To facilitate these operations, the code was developed following a modular design and only required the function calculating the new test metric to be provided. In the tutorial, we provide guidance on how to modify the functions to calculate custom testing metrics.

One of the challenges of using this database as benchmark is the potential data leakage problem. Data leakage occurs when information from the future is inadvertently used in model training, leading to overly optimistic performance estimates that won’t generalize to forecasts for future time periods. Users developing new models need to produce their forecasts in a pseudo-prospective fashion by training their models only on earthquakes occurred before 2008, and using only earthquakes that occurred before the starting day of each daily forecast to update. In general, users need to always ensure that training data precedes test data, and be cautious that statistical aggregations, derived variables, and transformations only incorporate data available at the forecasting time.

### Descriptive analysis

We first provide tools for a descriptive analysis of the forecasts. These include functions to visualize the temporal, spatial, and magnitude distributions of multiple models, compare them with observations, and create cumulative forecasts. Additionally, we provide functions to plot the temporal evolution of the cumulative and absolute daily number of expected earthquakes (Fig. 3 panel a, c, and d) for multiple models. The cumulative plot (panel a) also includes the observed number of earthquakes and therefore can be used to diagnose potential departures. The first absolute plot (panel c) compares the forecasted and observed daily number of events, and can be used to diagnose aftershock productivity, while the second one (panel d) shows the observations as points in a time-magnitude scatter plot and can be used to check if peaks in the number of events correspond to large magnitude earthquakes.

**Fig. 4:**
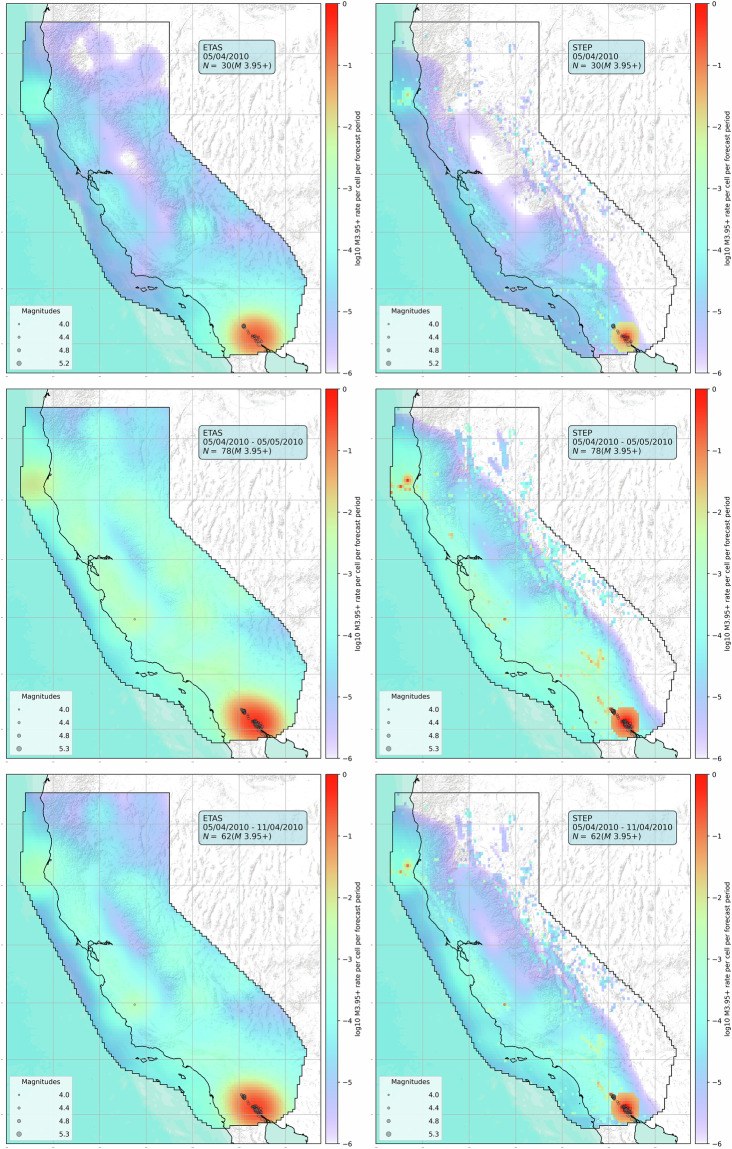
Spatial distribution of the expected number of *M* ≥ 3.95 earthquakes provided by the ETAS^31^ (left) and STEP^39^ (right) models. The forecasts from top to bottom refer to the day, week, and month after the *M*7.2 El Mayor-Cucapah earthquake occurred on April 4, 2010.

The code also enables building cumulative forecasts for custom time periods. The rates of a cumulative forecast are just the sum of the rates of the daily forecasts. These cumulative forecasts are useful to study the magnitude and spatial distributions of earthquake sequences. The code creates cumulative forecasts as pyCSEP GriddedForecast objects, and this is essential to utilize existing pyCSEP tools for visualisation, analysis, and evaluation. For example, we can use pyCSEP to plot their spatial distribution along with observations as shown in Fig. 4. We also provide a function to compare the magnitude distributions of cumulative forecasts with the observations (Fig. 3 panel b).

In the last section of the tutorial, we demonstrate the tools to convert forecasts in CSV file format to HDF5, along with an example, so that users can load their own forecasts and reuse the tools described above.

### Consistency with observations

**Fig. 5:**
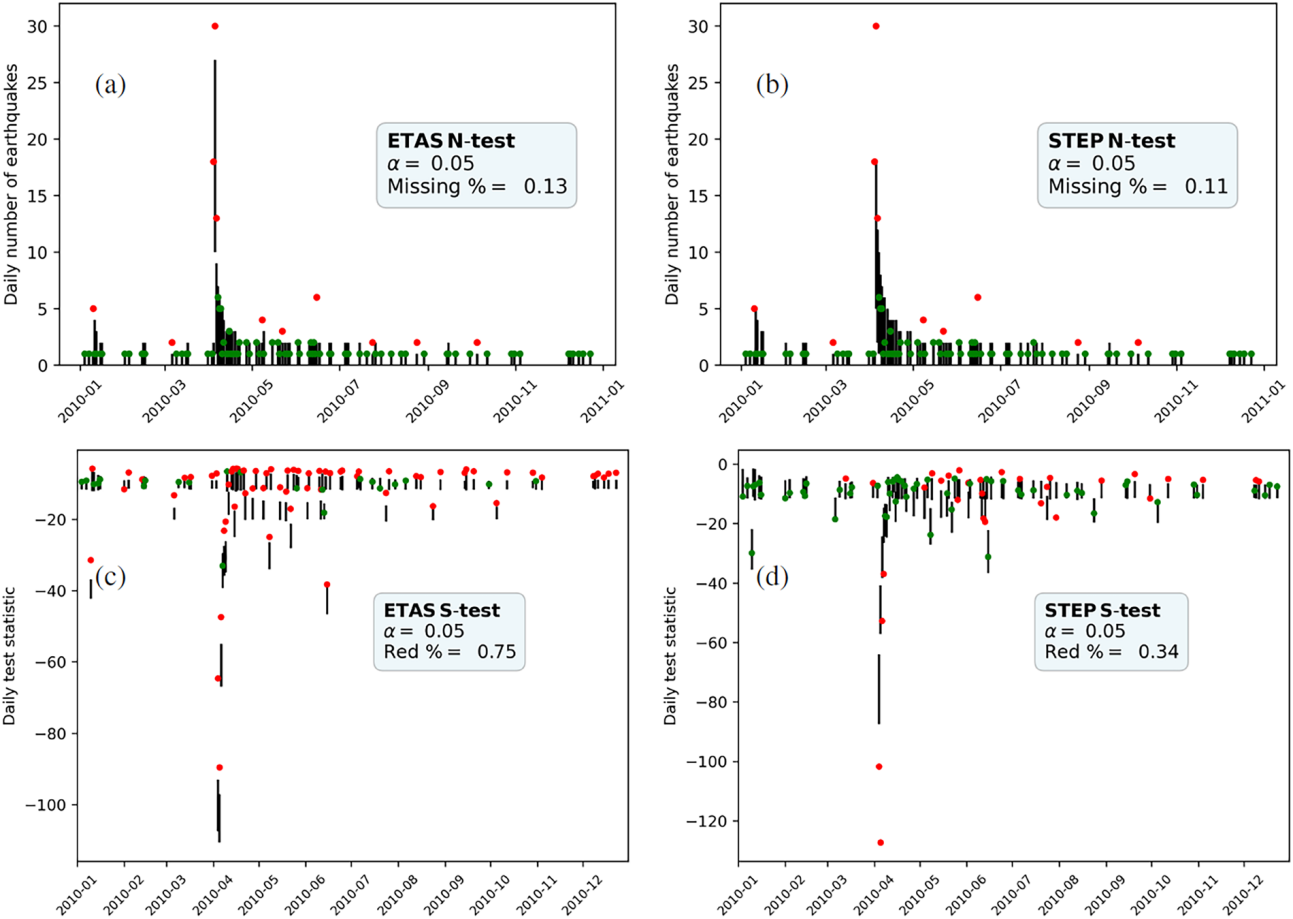
Results of CSEP tests, vertical lines represent the 95% interval of values expected by the model, red dots indicate days where the observed statistics is outside the interval indicating inconsistency between forecasts and observations, green dots the opposite. Each plot reports the name of the model and the test, the confidence level *α* at which the intervals are considered, and the percentage of days with observed statistic outside the interval, we expect this to be close to *α* for consistent forecasts. Panel (**a,b**) Daily N-test results for (respectively) the ETAS^31^, and the STEP^39^ models. Panel (**c**) and (**d**) Daily S-test results for (respectively) the ETAS and STEP models. The tests are computed on each day with at least one *M* ≥ 3.95 observed earthquake.

One of the accomplishments of CSEP is the development of a suite of likelihood-based consistency tests^24–26^, designed to evaluate grid-based forecasts against observed data on different aspects (e.g. total number of events, magnitude distribution, spatial distribution). These tests are available through the pyCSEP toolkit and can be easily computed on single days or cumulative forecasts in the GriddedForecast format. Here, we provide a function to calculate the test results on a daily basis given a set of forecasts. More specifically, the function requires a forecast in HDF5 format, an earthquake catalogue in pyCSEP format, and a test function from pyCSEP, and returns a table and a figure that illustrate the tests’ output. In this way, the function can be applied to new forecasting models and evaluation metrics. Figure 5 shows examples of output figures for the (number, panel a and b) N- and (spatial, panel c and d) S-test for the ETAS and STEP models. Red points indicate days where the observed statistics (the point) is outside the interval expected under the forecasting model (the vertical segments). In this way, it is easier to identify periods of time where a model is (in)consistent with the observations. The tutorial also provides guidance on how to interpret CSEP tests results. These tests are designed to evaluate the consistency of the forecasts with observed data and should not be used to compare models against each other.

### Comparison with other models

**Fig. 6:**
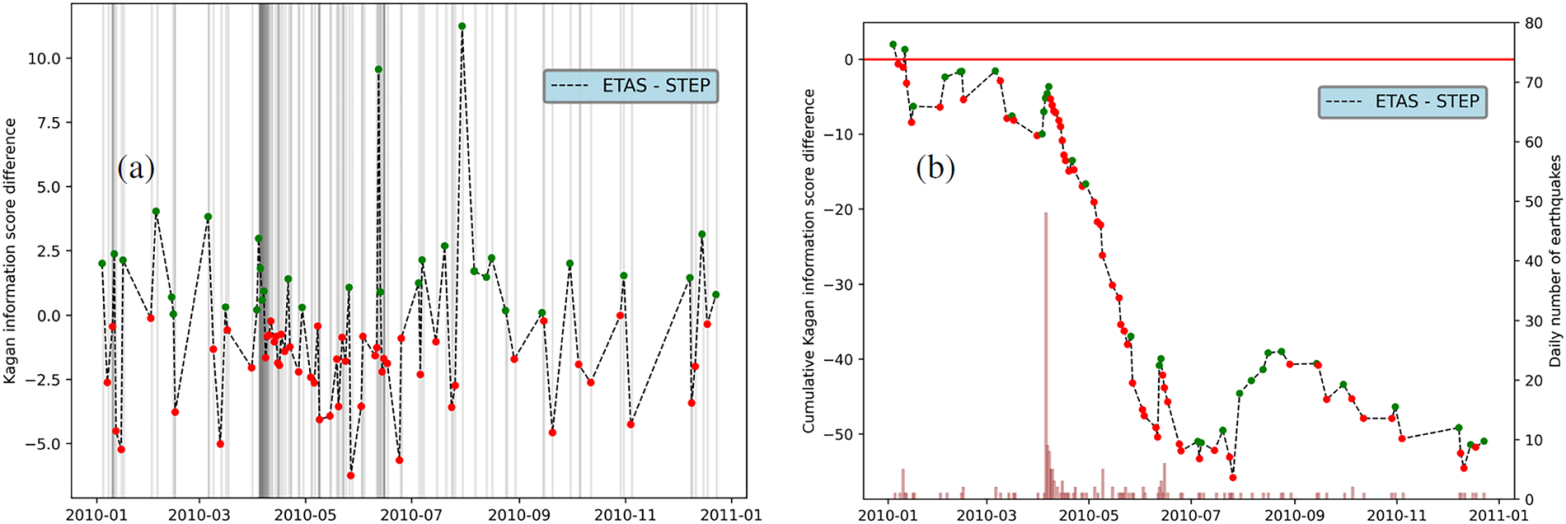
Temporal evolution of the Kagan information score difference between the ETAS^31^ and the STEP^39^ models. Green dots represent days with positive score difference (ETAS > STEP), while red dots indicate the opposite. Panel (**a**) Absolute Kagan information score differences. The vertical lines represents the occurrence time of observed *M* + 3.95 earthquakes. Panel (**b**) Cumulative Kagan information score differences. The histogram represents the daily number of observed *M* + 3.95 earthquakes. Frequencies are reported on the y-axis on the right side.

Along with consistency tests, CSEP developed a suite of comparative tests to assess the performance of alternative models based on the observations. This is done using standard statistical tests as a T-test of the log-likelihood differences^24,26^ or using scoring functions^48,49^. Here, we provide functions to calculate the Kagan information score^27^ for each day, and to visualise the temporal evolution of the absolute and cumulative differences (Fig. 6). The Kagan information score is a positively oriented score, i.e., the higher the better, and we can therefore interpret positive differences as favouring the ETAS model and negative ones to favour the STEP model. The cumulative plot highlights periods where a model is outperforming the other as shown in Fig. 6, where the STEP model consistently yields a higher score in the weeks after the El Mayor-Cucapah earthquake. The functions we provide only require as input a set of forecasts in HDF5 format and a catalogue of observed earthquakes in pyCSEP format so that they can be easily reused with new models and scores.

## Data Availability

All forecast HDF5 files along with the code and tutorial are openly accessible under the open access Creative Commons license. They can be downloaded from Zenodo^53^ (https://zenodo.org/records/15076187) where we provide a single zip folder with the forecasts, the code, and the tutorial. Alternatively, the files can be downloaded separately from the CSEP website (https://cseptesting.org/grid-based-forecasts/). The h5py^47^ Python package version 3.12.1 was used to create the HDF5 files from the original forecasts files stored in the testing center. The code and tutorial rely heavily on the pyCSEP^4–6^ toolkit to handle earthquakes forecasts and catalogues which is openly accessible on GitHub (https://github.com/SCECcode/pycsep). Additional pyCSEP tutorials can be found on the pyCSEP website (https://docs.cseptesting.org/tutorials/), while material from previous pyCSEP workshops is available on GitHub (https://github.com/cseptesting). The CSEP community have also developed a library to create reproducibility packages^50^ called floatCSEP^5,51^ also available on GitHub (https://github.com/cseptesting/floatcsep). The package can be used to create docker containers storing the code and data needed to reproduce the results reported in a publication. In this way, the interested reader only needs to execute the container to exactly reproduce all the figures in a manuscript. Examples are the reproducibility packages provided by Savran *et al*.^4^ and Graham *et al*.^5^ (respectively available at https://zenodo.org/records/6626265 and https://github.com/KennyGraham1/pyCSEP_followup_paper). We encourage the reader to use the database and code we provide in combination with floatCSEP to increase the reproducibility of the results.
